# Healthcare trajectories before and after critical illness: population-based insight on diverse patients clusters

**DOI:** 10.1186/s13613-019-0599-3

**Published:** 2019-11-09

**Authors:** Youenn Jouan, Leslie Grammatico-Guillon, Noémie Teixera, Claire Hassen-Khodja, Christophe Gaborit, Charlotte Salmon-Gandonnière, Antoine Guillon, Stephan Ehrmann

**Affiliations:** 1 Service de Médecine Intensive Réanimation, CHRU de Tours, 2 Bd Tonnellé, 37044 Tours Cedex 9, France; 2 INSERM U1100 Centre d’Etudes des Pathologies Respiratoires, Faculté de Médecine, Tours, France; 3 Université de Tours, Tours, France; 4 Service d’Information Médicale, d’Epidémiologie et d’Economie de la Santé, CHRU Tours, Tours, France; 5 INSERM CIC1415, CHRU Tours, Tours, France; 6 Service d’Accueil et d’Urgences, CHRU Tours, Tours, France; 7 CRICS-TriggerSep Research Network, https://www.triggersep.org

**Keywords:** Post-intensive care syndrome, Long-term outcome, Healthcare trajectories, Acute respiratory distress syndrome, Septic shock

## Abstract

**Background:**

The post intensive care syndrome (PICS) gathers various disabilities, associated with a substantial healthcare use. However, patients’ comorbidities and active medical conditions prior to intensive care unit (ICU) admission may partly drive healthcare use after ICU discharge. To better understand retative contribution of critical illness and PICS—compared to pre-existing comorbidities—as potential determinant of post-critical illness healthcare use, we conducted a population-based evaluation of patients’ healthcare use trajectories.

**Results:**

Using discharge databases in a 2.5-million-people region in France, we retrieved, over 3 years, all adult patients admitted in ICU for septic shock or acute respiratory distress syndrome (ARDS), intubated at least 5 days and discharged alive from hospital: 882 patients were included. Median duration of mechanical ventilation was 11 days (interquartile ranges [IQR] 8;20), mean SAPS2 was 49, and median hospital length of stay was 42 days (IQR 29;64). Healthcare use (days spent in healthcare facilities) was analyzed 2 years before and 2 years after ICU admission. Prior to ICU admission, we observed, at the scale of the whole study population, a progressive increase in healthcare use. Healthcare trajectories were then explored at individual level, and patients were assembled according to their individual pre-ICU healthcare use trajectory by clusterization with the K-Means method. Interestingly, this revealed diverse trajectories, identifying patients with elevated and increasing healthcare use (*n* = 126), and two main groups with low (*n* = 476) or no (*n* = 251) pre-ICU healthcare use. In ICU, however, SAPS2, duration of mechanical ventilation and length of stay were not different across the groups. Analysis of post-ICU healthcare trajectories for each group revealed that patients with low or no pre-ICU healthcare (which represented 83% of the population) switched to a persistent and elevated healthcare use during the 2 years post-ICU.

**Conclusion:**

For 83% of ARDS/septic shock survivors, critical illness appears to have a pivotal role in healthcare trajectories, with a switch from a low and stable healthcare use prior to ICU to a sustained higher healthcare recourse 2 years after ICU discharge. This underpins the hypothesis of long-term critical illness and PICS-related quantifiable consequences in healthcare use, measurable at a population level.

## Background

Survivors of critical illness frequently suffer from numerous sequelae, from physical and functional impairments to cognitive and psychiatric disorders, aggregated in the post intensive care syndrome (PICS) [1–3]. This syndrome has mostly been studied after sepsis/septic shock and acute respiratory distress syndrome (ARDS) [4–9]. Studies of healthcare use and costs after discharge from the intensive care unit (ICU) also showed an increased healthcare recourse and costs for ICU survivors [10–13]. However, this increased healthcare use after critical illness has been mainly compared to “non-ICU cohorts” [10, 13, 14] and differences in case-mix preclude any conclusions regarding potential effects of critical illness itself on post intensive care healthcare use. Indeed, the causal association between ICU stay and subsequent disabilities and morbidity is challenging to investigate, as critical illness and so-called PICS may share common risk factors and triggers, rooted in patients’ healthcare trajectories prior to ICU admission. Studies of long-term outcome after critical illness frequently reported comorbidities as a strong determinant of long-term death [15–17], and comorbidities are very prevalent among patients admitted to ICU [18, 19]. Thus, the impact, on post-ICU healthcare trajectories, of pre-existing heterogeneity before ICU admission regarding healthcare use and comorbidities has not been finely assessed. Therefore, it is still uncertain to which extent critical illness per se is responsible for prolonged increased healthcare use after ICU, or to which extent pre-existing comorbidities and pre-ICU healthcare trajectories impact the post-ICU burden.

To explore this question, we assessed the healthcare use trajectories prior to ICU admission and posterior to ICU discharge using the exact same methodology, among a cohort of ICU survivors.

## Methods

### Study population

**Fig. 1:**
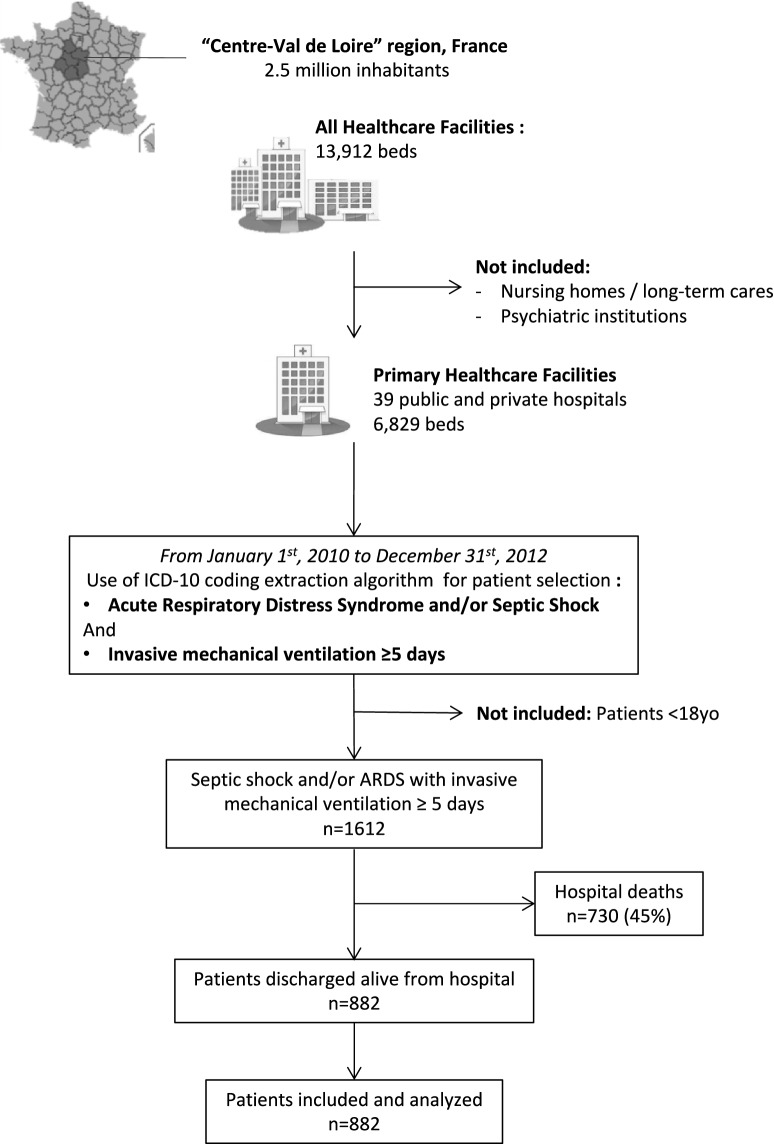
Flowchart describing data extraction and patients’ retrospective process from the database, during the study period. *ARDS* acute respiratory distress syndrome

At the population level of one French region, all adult patients admitted to an ICU from January 1st, 2010 to December 31st, 2012, for septic shock and/or ARDS, with 5 days or more invasive mechanical ventilation, were extracted from the regional medico-administrative database (“*Programme de Médicalisation des Systèmes d’Information*”, PMSI) using a computerized algorithm. This database relies on the mandatory notification of each hospital stay, through a coded summary, for all public and private French hospitals. Every hospital stay in the database is linked to patient data using an encrypted anonymized number, allowing to carry out epidemiological analyses among a comprehensive historical cohort. The study was performed in one representative French region (Centre Val de Loire, 2.5 million inhabitants), including one university hospital, one tertiary hospital and 37 general and private hospitals. Eight hospitals of the region have at least one intensive care unit (Fig. 1).

Algorithm selection criteria were (see Additional file 1: Table S1, Additional file 2: Table S2):

- Presence of “ARDS” and/or “Septic shock” codes from the International Classification of Disease, Tenth Revision (ICD-10), as primary or secondary diagnosis.
- Invasive mechanical ventilation reported for 5 days or more, using the corresponding codes from the French Common Classification of Medical Acts.

Patient under 18 years and pediatric ICUs were not included.

Performance of the selection algorithm was validated by a blinded review of medical charts of 180 patients randomly selected, comprising 70 cases selected by the inclusion algorithm and 110 controls, from two different hospitals and admitted in ICU during the study period and who did not met criteria of the selection algorithm. Investigators then blindly reviewed medical chart for case validation. ARDS was defined according to the Berlin Definition [20], and septic shock was defined as the presence of a suspected or proven infection associated with hypotension requiring vasopressive therapy after adequate fluid resuscitation (as mentioned in the medical chart, and/or at least 30 mL/kg). Invasive mechanical ventilation was defined by mechanical ventilation through a tracheal tube or tracheostomy. Algorithm performance appeared excellent with a positive predictive value of 96%, and a negative predictive value of 92%.

The purpose of the study was to explore post-ICU burden in terms of healthcare use; thus, among ICU patients identified by the algorithm, only patients discharged alive from the hospital were included in the study cohort.

### Data collection

Index hospitalization was defined as the whole acute care hospital stay during which the index ICU stay occurred. If a patient had two or more hospitalizations satisfying inclusion criteria, the first hospitalization was considered as the index hospitalization. Age, sex, simplified acute physiology score (SAPS) 2, duration of mechanical ventilation, length of stay in the ICU and overall hospital length of stay were collected for each index hospitalization. Patients were tracked during a 2-year period before and a 2-year period after the index ICU hospitalization (which are, respectively, termed “pre-ICU period” and “post-ICU period” thereafter).

Comorbidities were retrieved for each patient. For this purpose, we extracted ICD-10 coding related to chronic comorbidities and grouped them into categories according to the Charlson Comorbidity Index [21] (Additional file 3: Table S3). Comorbidity-related ICD-10 codes were then tracked during the pre-ICU and the post-ICU periods to build pre-ICU and post-ICU comorbidity reports. Mortality at hospital during the post-ICU follow-up was also recorded.

Healthcare use was similarly analyzed during pre-ICU and post-ICU period using the exact same methodology for data extraction. Specifically, we extracted the number of days in hospitalization and the number of ambulatory care/consultations (considered as 1-day healthcare use). We also extracted the type of healthcare use: acute care settings (either medical or surgical), rehabilitation centers, psychiatry, or “hospitalization at home” (a specific setting with high intensity care and nursing organized at the patient’s home).

### Analyses

To assess consequences of active medical conditions in terms of healthcare use, we specifically focused on the recourse to acute care facilities, either for complete hospitalizations (at least one overnight stay) or for ambulatory hospitalization (full day spent in hospital for multiple consultations, interventions, and treatment) that we designated as “days of healthcare use”. Thus, hospitalization in rehabilitation centers and simple ambulatory consultations were not included in this definition.

Temporal trends of healthcare use analyses were performed by quarterly assessing frequency of days of healthcare use (% of days), calculated as the quarterly report of the numbers of days of healthcare use divided by the number of patient-days during the quarter. For instance, a patient who would have spent 5 days at a hospital, and then 4 ambulatory hospitalizations during a quarter would be considered as having 9 days/90 days = 10% of days spent for healthcare use. This procedure enables to take into account patients lost for follow-up and deaths in the post-ICU period.

The main hypothesis underlying the present work was that the population of patients admitted in ICU for ARDS/septic shock would have heterogeneous healthcare trajectories prior to ICU admission, and thus, further analysis of post-ICU healthcare use should integrate this parameter.

To do so, we had to group patients according to (1) their level of pre-ICU healthcare use, and (2) the temporal dynamic of this pre-ICU healthcare use. Practically, this could be done by clustering patients according to quarterly evaluation of healthcare use in pre-ICU period. We used the K-Means clustering technic, allowing an unsupervised and unbiased approach to aggregate patients together based on their similarity in healthcare use for each quarter in pre-ICU period. For this purpose, and to capture all aspects of healthcare use, we used a broader definition of healthcare use for the clustering, encompassing recourse of hospitalizations, ambulatory consultations, in acute care settings and rehabilitation centers. We tested the algorithm with a number of clusters ranging from 2 to 10 (i.e., *K* from 2 to 10) and analyzed for each test the percentage of inter-group variance explained by constitutions of the groups. When increasing the number of clusters (i.e., increasing the value of *k*), the inter-group variance explained by those clusters likely increases. Thus, the objective of this method is to reach a reasonably high inter-group variance explained by constitution of the clusters (i.e., close to 70%), with a clinical relevant number of clusters. At the end, in our dataset, a *K* = 5 led to a good variance (67%) along with a relevant number of clusters to analyze, and with no further significant increase in inter-group variance for *K* > 5. Once clusters of patients were built, we could next plot the quarterly healthcare use—in both pre- and post-ICU periods—for each cluster to build pre- and post-ICU healthcare trajectories in the different clusters.

For pre-post ICU periods comparisons, Wilcoxon and McNemar tests for paired data were used, as appropriate.

For comparisons between groups, Chi-square and ANOVA tests were used, as appropriate.

Kaplan–Meier survival curve analysis was used to explore mortality during the post-ICU period.

Statistical analyzes were performed with SAS version 9.4 (SAS Institute Inc., Cary, NC) and R 3.2.2 (https://www.R-project.org). A *p* < 0.05 was as considered statistically significant.

## Results

### Description of the population

During the study period, 1612 patients were identified by the algorithm, of whom 730 (45%) died during the index hospitalization. Thus, 882 patients who were discharged alive from the hospital were included in the study and analysed (Fig. 1). Characteristics of the ICU patient stays are presented in Table 1. During the pre-ICU period, 310 patients (35%) had at least one main chronic organ disease among cardiac, renal, respiratory and hepatic functions reported. Moreover, cancer was reported in 130 patients (14.7%), obesity in 95 patients (10.8%) and alcohol abuse in 85 patients (9.6%). Conversely, 47% of the patients (*n* = 418) had no comorbidities reported during the pre-ICU period. Compared to the pre-ICU period, we observed an increased report of chronic cardiac, respiratory and renal diseases, in the post-ICU period (Table 2). During the 2-year follow-up, hospital mortality was 15.5% (Additional file 4: Figure S1 shows Kaplan–Meier curve of cumulative probability of survival).

**Table 1:** Patient characteristics

Variables	
Patients discharged alive from the hospital and included, n	882
Age, mean ± SD	61 ± 15
Sex ratio, male/female	1.9
Diagnosis, n (%)	
ARDS	310 (35)
Septic shock	444 (50)
ARDS and septic shock	128 (15)
SAPS2, mean ± SD	49 ± 17
Duration of invasive mechanical ventilation (days), median (IQR)	11 (8, 20)
Length of stay (days), median (IQR)	
ICU	19 (12; 33)
Hospital	42 (29; 64)

*ARDS* acute respiratory distress syndrome, *ICU* intensive care unit, *SAPS2* simplified acute physiology score 2, *SD* standard deviation, *IQR* interquartile range (25th and 75th percentiles)

**Table 2:** Comparison of the reported comorbidities related to chronic organ dysfunction during the 2-years pre-ICU and two years post-ICU period, for the whole population (*n* = 882)

Comorbidity	Pre-ICU *n*, (%)	Post-ICU *n*, (%)	*p*
Chronic cardiac disease	140 (16)	220 (25)	< 0.001
Chronic respiratory disease	79 (9)	118 (13)	< 0.001
Chronic renal disease	46 (5)	87 (10)	< 0.001
Chronic hepatic disease	45 (5)	55 (6)	ns

*ns* non-significant

### Global analysis of healthcare use trajectories prior and posterior to the index ICU stay

**Fig. 2:**
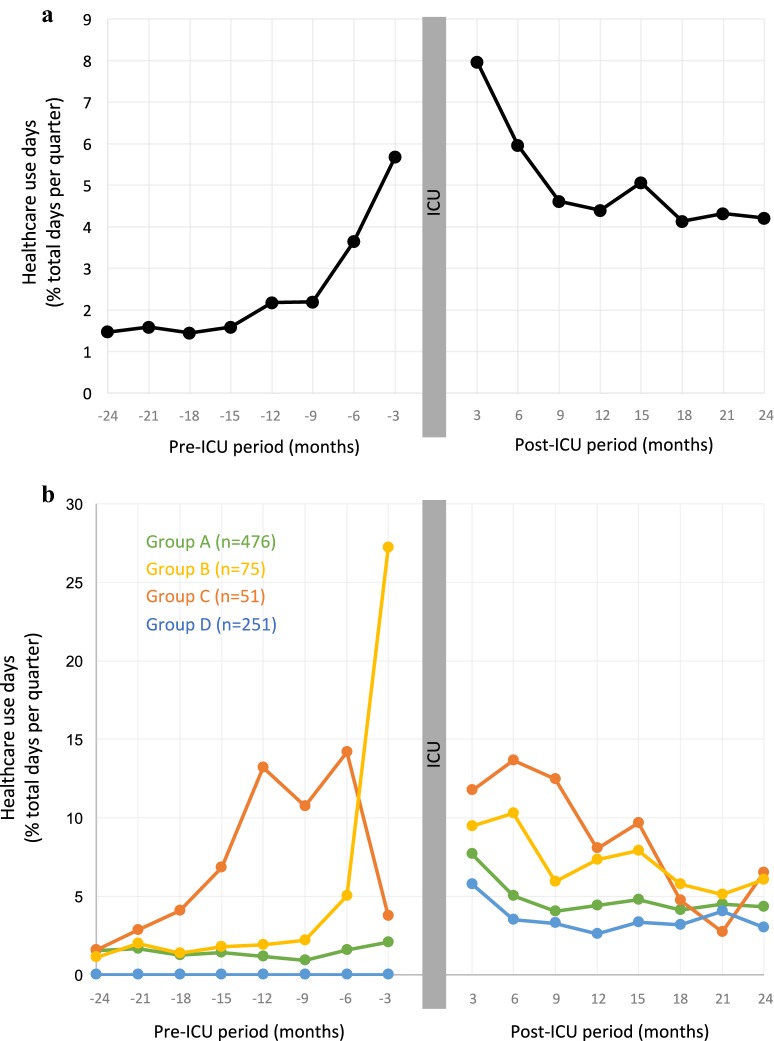
Healthcare use days (% of total days per quarter spent in acute care settings) during the pre-ICU period and the post-ICU period, **a** for the whole population, and **b** after clustering based on total pre-ICU healthcare use (see text fort details regarding clustering method)

Figure 2a shows the trends in healthcare use during the pre-ICU period and the post-ICU period. During, the pre-ICU period, we first observed an overall relatively low and stable healthcare use 2 years before the ICU stay (mean percentage of healthcare use: 1.3 ± 3.1%), followed by a gradual increase in healthcare use the year before the index hospitalization. During the post-ICU period, the highest level of healthcare use occurred during the first 6 months post-ICU. Thereafter, the proportion of healthcare use days decreased and seemed stable at the end of the follow-up, but remained at a higher level than the pre-ICU period (mean percentage of healthcare use: 4.5 ± 8.6%).

### Cluster analysis of healthcare use trajectories (Fig. 2b)

Using each patients’ individual healthcare use during the pre-ICU period, five clusters were built using the K-Means method (see “Methods” section). Among them, two clusters with a reduced number of patients (12 and 14 patients) were observed, which could not be integrated in other clusters, even when reducing *K* value. These two small groups represented patients with a very high pre-ICU healthcare use (mean percentage of healthcare use days of 16.6 ± 12.9% and 11.6 ± 6.4%, respectively, compared to 1.3 ± 3.1% for the whole population), with important specific major comorbidities (e.g., start of chronic dialysis during the pre-ICU period). These two groups, representing a total of 26 patients (3% of the overall study population), were considered as outliers and not analyzed further. Three clusters remained, with one constituted of 54% of the study population (*n* = 476 patients) and thereafter named group A. This cluster could not be more finely clustered by increasing *K* above 5, indicating a strong intra-group homogeneity. The two remaining clusters consisted of 75 and 51 patients, respectively, named group B and group C. Last, patients who had no healthcare use during the whole pre-ICU period, could not, by definition, be included in the pre-ICU healthcare-use-based K-Mean clustering, and constituted a group of 251 patients, named group D. The main characteristics of the 4 groups regarding pre-ICU comorbidities and index hospitalization are shown in Table 3. Interestingly, group A had significantly less comorbidities reported during the 2-year period in pre-ICU, while simplified acute physiology score II (SAPSII), duration of mechanical ventilation, and ICU and hospital length of stay were not significantly different across the four groups (Table 3).

**Table 3:** Patient characteristics in groups according to the 2-year-period pre-ICU healthcare use: groups A, B and C built after clustering (*see text for details*), and group D who had no pre-ICU healthcare use reported

Variables	Group A (*n* = 479)	Group B (*n* = 75)	Group C (*n* = 51)	Group D (*n* = 251)	*p*
Percentage of total study population	54.3	8.5	5.8	28.5	
Age, mean ± SD	61 ± 15	63 ± 14	62 ± 15	61 ± 15	0.8
Main comorbidities reported (during 2-year pre-ICU period)					
Chronic cardiac disease, *n* (%)	82 (17.1)	23 (30.6)	21 (41)	NA	< 0.001*
Chronic respiratory disease, *n* (%)	49 (10.2)	13 (17.3)	13 (25.4)	NA	0.003*
Chronic hepatic disease, *n* (%)	22 (4.5)	14 (6, 14)	9 (17.6)	NA	< 0.001*
Chronic renal disease, *n* (%)	17 (3.5)	14 (18.6)	6 (11.7)	NA	< 0.001*
SAPSII, mean ± SD	49 ± 17	53 ± 18	50 ± 19	49 ± 17	0.78
Duration of mechanical ventilation (days), mean ± SD	17 ± 15	13 ± 10	15 ± 14	15 ± 11	0.10
ICU LOS (days), mean ± SD	27 ± 22	21 ± 16	26 ± 22	26 ± 19	0.23
Hospital LOS (days), mean ± SD	53 ± 37	52 ± 48	59 ± 46	47 ± 25	0.18

*SAPSII* simplified acute physiology score II of the index intensive care unit (ICU) stay, *SD* standard deviation, *LOS* length of stay, *NA* non applicable

* Tests performed between groups A, B and C

Time trends analyses of quarterly healthcare use days for the 4 groups, in both pre-ICU and post-ICU periods, are represented in Fig. 2b. Group A (n = 476) had a mean healthcare use days during the pre-ICU period of 1.4 ± 1.9%, which appeared to be stable over time, across the whole pre-ICU period. Interestingly, during the post-ICU period, this group switched to a higher level of healthcare use (mean of 5.0 ± 7.0% of healthcare use days), that appeared to be sustained and stable from 6 months post-ICU until the end of the 2-year follow-up (Fig. 2b). Group B and C displayed different patterns of healthcare use in the pre- and post-ICU periods. First, considering the whole pre-ICU period, group B and C had a higher mean healthcare use days compared to group A (respectively 5.3 ± 2.6% and 7.2 ± 3.5% healthcare use of days). Regarding temporal dynamics of their healthcare use, group B displayed a steep slope of increase in the 6 last months before ICU admission, and group C displayed a sustained increase in healthcare use days over 18 months prior to ICU admission, but group C specifically displayed a further decline prior to ICU admission (Fig. 2b). This decrease in healthcare use before ICU was consistently found across healthcare resources involved (hospitalizations, ambulatory care, admission to emergency departments) and was not explained by increase in other healthcare resources not included in this graphical representation: rehabilitation centers, ambulatory consultations, or “hospitalization at home” (data not shown). Regarding post-ICU healthcare use, groups B and C also displayed a higher level of healthcare use compared to the pre-ICU period (mean of 7.5 ± 6.7% and 9.4 ± 11.4% of healthcare use days, respectively), mainly during the first-year post-ICU (Fig. 2b). Last, group D, constituted of patients who had no healthcare use recorded during the pre-ICU period (*n* = 251), displayed a similar profile to group A in the post-ICU period, with a persistent high healthcare use (3.7 ± 6.4% healthcare use days over the two post-ICU years) that also appeared stable and sustained from 6 months post-ICU to the end of the 2-year follow-up (Fig. 2b).

## Discussion

In this population-based study, we comprehensively tracked healthcare use of ICU survivors admitted for ARDS and/or septic shock ventilated at least 5 days, 2 years before and 2 years after ICU. Using a clustering strategy based on pre-ICU healthcare use, we could depict the different healthcare trajectories nested in the global population of ICU survivors. Global pre-ICU healthcare trajectory revealed a progressive increase in healthcare use, but clustering analyses showed that most of the patients had a stable low healthcare use until ICU admission or no healthcare recourse.

Interestingly, Szakmany et al. [17], in a recent population-based data linkage study, similarly reported a high healthcare facility use the year before ICU admission, and Lone et al. [10] also observed in their population-based study a global increase in healthcare use before ICU admission. Hence, one may consider that acute critical illness is the consequence of a global worsening medical condition, and consequently, post-ICU increased healthcare use and PICS might be its consequences rather than causally related to the acute critical illness and ICU stay per se. The present work identifies various trajectories hidden in this global picture. The methodology of the present work, using the exact same methods to assess healthcare use of the pre- and post-ICU periods, enabled a paired analysis, each patient being its own control. Thus, patients with low and stable or without any pre-ICU healthcare use, which represented the vast majority of patients (83%), had a persistent and stable increased healthcare use over the 2 years post-ICU. This suggests that the acute critical illness episode had a key role in the healthcare use trajectory of most survivors who have moderate and stable healthcare use before, resulting in a higher level of “basal” healthcare use after the ICU stay. These results could be regarded as measurable consequences of PICS at the population level. Moreover, major comorbidities related to renal, respiratory and cardiac functions were more frequently reported in the post-ICU period compared to the pre-ICU period, also supporting this view. Regarding the group of patients with elevated healthcare use in pre-ICU period, our clustering analysis identified two groups with different dynamic of healthcare use utilization. Thus, group B displayed a steep increase before ICU admission, probably as a result of worsening underlying medical conditions, eventually leading to acute critical illness. Group D, however, displayed a decrease before ICU admission, which appears unexpected following a period of high healthcare use. We cannot rule out the possibility—although unlikely—that these patients were referred to other health structures outside of the study region, or referred only to private ambulatory physicians unrelated to private hospitals, both being uncaptured by our extraction algorithm. We can also hypothesize that it represented patients lost for follow-up after an important period of healthcare use, and consequently readmitted later in critical condition. Underpinning this hypothesis, this group had a large proportion of patients with chronic respiratory and cardiac diseases, medical conditions for which close follow-up is mandatory to avoid unscheduled emergency readmissions [22, 23].

These results are of interest in the current new era of critical care providers’ concern about long-term outcome of their patients: a better profiling and understanding of healthcare use trajectories of critically ill patients will help understand patients most likely to benefit of ICU admission in the long term and give valuable information for setting up follow-up programs. Interestingly, differences in healthcare use observed in pre-ICU among the five groups remained consistent in post-ICU period. Moreover, despite these important differences in level of healthcare use, there were no obvious differences in main characteristics of the ICU stay between groups (Table 3). In the same line, Lone et al. [10] showed that prior illness and healthcare use were stronger predictor of post-ICU hospital readmissions than the acute critical illness by itself. Thus, such pre-ICU healthcare trajectories analyses may be of interest in future works to improve long-term outcome of critically ill patients. Integrating pre-ICU healthcare trajectories to select patients for post-ICU follow-up programs could, therefore, be helpful and more discriminant than characteristics of the ICU stay itself. Moreover, we might hypothesize that dedicated post-ICU follow-up interventions will differentially affect patients, depending on their specific healthcare use trajectory prior to ICU admission. Specifically, targeting patients with a previous stable and low healthcare for post-ICU follow-up might be more efficient, with recovery objectives easier to define.

At a public health level, for the evaluation of global ICU-induced costs, this work paves the ground for future detailed analyses and evaluations according to healthcare use trajectories. Previous landmark studies have demonstrated elevated costs associated with ICU admissions, compared to non-ICU control populations [10, 24]; however, differences in case-mix preclude detailed analysis and interpretation. Our work shows that relevant sub-groups among ICU patients with prior moderate and stable healthcare use could be defined, and be relevant to settle global pre-ICU, ICU and post-ICU healthcare costs, enabling comprehensive cost/effectiveness assessment of ICU admission and potential post-ICU follow-up programs.

Last, at a conceptual level, the observed switch in healthcare use trajectories after critical illness, with a persistent increased healthcare use after critical illness can also be analysed using the theoretical frameworks of the critical transitions and loss of resilience, that has recently gained considerable interest in many science fields, including systems biology [25, 26]. One could, thus, interpret critical illness as the tipping point, with further frailty and low resilience state during the post-ICU period, with measurable consequences in terms of frequent healthcare use.

This study has several limitations. First, we used administrative databases which could be at risk for miscoding or undercoding. The French national hospital database used here was initially designed for billing purposes but now appears to be a powerful tool for epidemiological surveillance on the condition that the selection algorithm was validated [27–30]. We assessed the performance of our selection algorithm through blinded review of medical charts randomly selected and we observed a very good performance of the detection (positive predictive value 96%, negative predictive value of 92%). Second, during the post-ICU period, patients who were lost to follow-up and who died might bias data interpretation. However, we aimed at limiting this bias (1) by restricting our analyses to patients living in the region during the study period, and, thus, unlikely to have healthcare use outside the region—not captured in the study and (2) by using quarterly measurements of healthcare use reported as a ratio on the total days spend alive with follow-up for each patient considered analysed. Third, data extraction was limited to hospitals (public and private); thus, healthcare use associated with individual practitioners not affiliated with a hospital was not evaluated in our study, leading to a possible under-estimation of healthcare use and/or lack of capture of associated specific healthcare use trajectories. However, one may assume that the most severe cases of healthcare recourse lead to hospitalization at one point, or, at least, an ambulatory care/specialist consultation affiliated with a hospital, all captured in the present study. Last, we could not extract data regarding frailty, which has recently emerged as an important and relevant concept in critical care [31, 32].

## Conclusions

In this study, we comprehensively tracked healthcare use of a population of patients, 2 years before and 2 years after their ICU stay for septic shock and/or ARDS with the exact same methodology, each patient being its own control. Clustering analysis identified an important sub-group of patients with a low and stable pre-ICU healthcare use until ICU admission. This group displayed a higher and stable healthcare use 2 years after ICU discharge, suggesting a pivotal role of critical illness in patients’ healthcare use trajectories. Based on these results, PICS seems to have measurable consequences at a population level that could be used to design effective interventions aiming at reducing this post-intensive care burden of care.

## Supplementary information

**Additional file 1: Table S1.** Case definition for patient selection using primary and secondary diagnoses and procedure codes in discharge summaries.

**Additional file 2: Table S2.** Codes from the 10th edition of the International Classification of Disease (ICD-10) used for definition of septic shock, acute respiratory distress syndrome (ARDS) and sepsis.

**Additional file 3: Table S3.** Codes from the 10th edition of the International Classification of Disease (ICD-10) used for defining comorbidities derived from Charlson Comorbidity index.

**Additional file 4: Figure S1.** Kaplan–Meyer curve of cumulative survival rate during the 2-years after intensive care unit admission, starting at hospital discharge. Gray area: confidence interval at 95%.

## Data Availability

Data are available from the authors upon reasonable request and with the permission of the institution.

## Abbreviations

ARDS: acute respiratory distress syndrome
ICU: intensive care unit
ICD-10: International Classification of Diseases, 10th edition
IQR: interquartile range
PICS: post intensive care syndrome
SAPS2: simplified acute physiology score 2
SD: standard deviation
