# Supplementary material for: Healthcare trajectories before and after critical illness: population-based insight on diverse patients clusters
Source: Ann Intensive Care. 2019 Nov 9;9:126. doi: 10.1186/s13613-019-0599-3 (PMC6842359; doi:10.1186/s13613-019-0599-3)
Supplement: Supplementary file 1 — Additional file 1: Table S1. Case definition for patient selection using primary and secondary diagnoses and procedure codes in discharge summaries. [file 13613_2019_599_MOESM1_ESM.docx]

**Additional File 1: Table S1 –** Case definition for patient selection using primary and secondary diagnoses and procedure codes in discharge summaries.

|  | Hierarchy in the discharge summary | | |
| --- | --- | --- | --- |
|  | Primary Diagnosis | Secondary Diagnosis | Procedure code |
| Case definition | Septic Shock | - | Mechanical ventilation >5days |
|  | ARDS | - |  |
|  | Sepsis | Septic Shock and/or ARDS |  |

ARDS: acute respiratory distress syndrome
