# Supplementary material for: Healthcare trajectories before and after critical illness: population-based insight on diverse patients clusters
Source: Ann Intensive Care. 2019 Nov 9;9:126. doi: 10.1186/s13613-019-0599-3 (PMC6842359; doi:10.1186/s13613-019-0599-3)
Supplement: Supplementary file 2 — Additional file 2: Table S2. Codes from the 10th edition of the International Classification of Disease (ICD-10) used for definition of septic shock, acute respiratory distress syndrome (ARDS) and sepsis. [file 13613_2019_599_MOESM2_ESM.docx]

| **Septic Shock** | R572 |
| --- | --- |
| **ARDS** | J80 |
| **Sepsis** | A010, A021, A022, A150, A151, A152, A153, A160, A161, A162, A202, A209, A212, A217, A219, A239, A241, A244, A282, A310, A319, A370, A371, A378, A379, A394, A399, A400, A401, A402, A403, A408, A409, A410, A411, A412, A413, A414, A415, A419, A420, A427, A429, A430, A439, A481, A490, A491, A492, A493, A498, A499, A500, A527, A548, A549, A698, A699, A70, A78, A798, A799, B012, B052, B068, B200, B206, B250, B334, B340, B341, B342, B348, B371, B377, B379, B380, B381, B382, B389, B390, B391, B392, B400, B401, B402, B409, B410, B419, B420, B429, B440, B441, B449, B450, B459, B460, B469, B49, B583, B589, B59, B664, B671, B75, B778, B779, B950, B951, B952, B953, B954, B955, B956, B957, B958, B960, B961, B962, B963, B964, B965, B968, B970, B971, B972, B974, B978 |

**Additional File 2: Table S2** – Codes from the 10^th^ edition of the International Classification of Disease (ICD-10) used for definition of septic shock, acute respiratory distress syndrome (ARDS) and sepsis
