# Supplementary material for: Healthcare trajectories before and after critical illness: population-based insight on diverse patients clusters
Source: Ann Intensive Care. 2019 Nov 9;9:126. doi: 10.1186/s13613-019-0599-3 (PMC6842359; doi:10.1186/s13613-019-0599-3)
Supplement: Supplementary file 3 — Additional file 3: Table S3. Codes from the 10th edition of the International Classification of Disease (ICD-10) used for defining comorbidities derived from Charlson Comorbidity index. [file 13613_2019_599_MOESM3_ESM.docx]

| **Comorbidity** | **Related ICD-10 codes** |
| --- | --- |
| Chronic heart disease | A520, I05-I08, I091, I098, I099, I110, I130, I132, I255, I34-I39, I420, I425-I429, I43, I441-I443, I456, I459, I47-I49, I50, P290, Q230-Q233, R000, R001, R008, T821, Z450, Z950, Z952-Z954, I21, I22, I252 |
| Chronic pulmonary disease | I278, I279, J40-J47, J60-J67, J684, J701, J703 |
| Chronic renal disease | I120, I131, N032-N037, N052-N057, N18, N19, N250, Z490-Z492, Z940, Z992 |
| Chronic liver disease | B18, I85, I864, I982, K70, K711, K713-K715, K717, K72-K74, K760, K762-K769, Z944 |
| Hypertension | I10-I13, I15 |
| Uncomplicated diabetes | E100, E101, E109, E110, E111, E119, E120, E121, E129, E130, E131, E139, E140, E141, E149 |
| Complicated diabetes | E102-E108, E112-E118, E122-E128, E132-E138, E142-E148 |
| Pulmonary circulation disorders | I26, I27, I280, I288, I289 |
| Peripheral vascular disorders | I70, I71, I731, I738, I739, I771, I790, I792, K551, K558, K559, Z958, Z959 |
| Chronic rheumatoid disease | L940, L941, L943, M05, M06, M08, M120, M123, M30, M310-M313, M315, M32-M35, M360, M45, M461, M468, M469 |
| Neurological disease | F00-F03, F051, G041, G114, G30, G311, G10-G13, G20-G22, G254, G255, G312, G318, G319, G32, G35-G37, G40, G41, G45, G46, G801, G802, G81, G82, G830-G834, G839, G931, G934, H340, I60-I69, R470, R56 |
| AIDS/HIV | B20-B22, B24 |
| Cancer | C00-C26, C30-C34, C37-C41, C43, C45-C58, C60-C85, C88, C900, C902, C96, C97 |
| Immunological deficiency syndrome | D80-D84, Z9480 |
| Chronic anemia | D500, D508, D509, D51-D53 |
| Coagulopathy | D65-D68, D691, D693-D696 |
| Obesity | E66 |
| Hypothyroidism | E00-E03, E890 |
| Fluid and electrolyte disorders | E222, E86 E87 |
| Psychiatric disorder | F20, F22-F25, F28, F29, F302, F312, F313-F315, F32, F33, F341, F412, F432 |
| Drug abuse | F11-F16, F18, F19, Z715, Z722 |
| Alcohol abuse | E52, F10, G621, I426, K292, K700, K703, K709, T51, Z502, Z714, Z721 |

**Additional File - Table S3** – Codes from the 10^th^ edition of the International Classification of Disease (ICD-10) used for defining comorbidities derived from Charlson Comorbidity index

AIDS : Acquired Immunodeficiency Syndrome; HIV: human immunodeficiency virus
