# Supplementary material for: Healthcare trajectories before and after critical illness: population-based insight on diverse patients clusters
Source: Ann Intensive Care. 2019 Nov 9;9:126. doi: 10.1186/s13613-019-0599-3 (PMC6842359; doi:10.1186/s13613-019-0599-3)
Supplement: Supplementary file 4 — Additional file 4: Figure S1. Kaplan–Meyer curve of cumulative survival rate during the 2-years after intensive care unit admission, starting at hospital discharge. Gray area: confidence interval at 95%. [file 13613_2019_599_MOESM4_ESM.pdf]

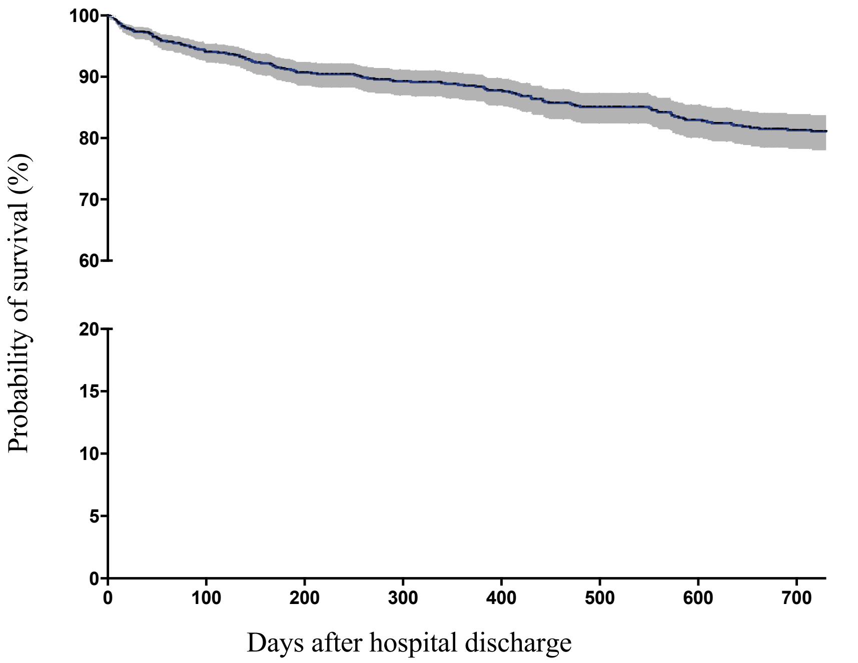

Additional File 4: Figure 1 – Kaplan-Meyer curve of cumulative survival rate during the two-years after intensive care unit admission, starting at hospital discharge. Gray area : confidence interval at 95%
